# Supplementary material for: Boosting Mitochondrial Biogenesis Diminishes Foam Cell Formation in the Post-Stroke Brain
Source: Int J Mol Sci. 2023 Nov 23;24(23):16632. doi: 10.3390/ijms242316632 (PMC10706318; doi:10.3390/ijms242316632)
Supplement: Supplementary file 1 [file ijms-24-16632-s001.zip › ijms-2710076-supplementary.pdf]

Supplementary Figures

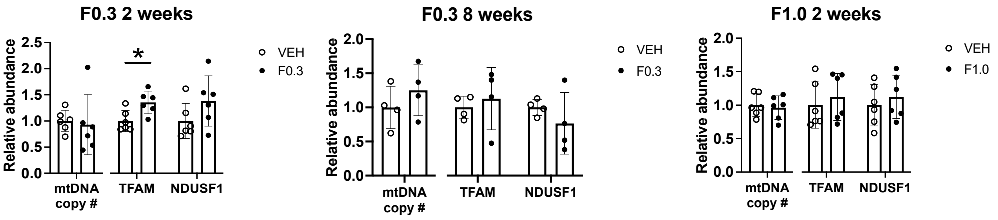

Supplementary Figure S1. Mitochondrial marker levels did not differ significantly between the treatment groups on the contralateral side, except for TFAM, which was elevated in mice treated with formoterol 0.3 mg/kg for 2 weeks. t(9.935)=2.940, *p=0.0149, unpaired two-tailed t-test with Welch's correction. n=4-6. Data presented as mean ± SD.

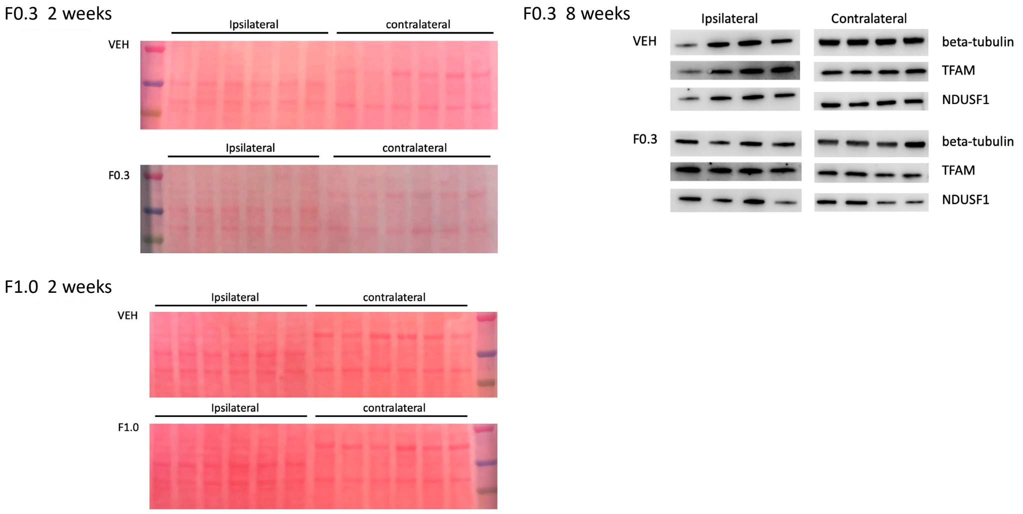

Supplementary Figure S2. Original images of Western Blots.
